# Datasets on the optimization of alginate extraction from *sargassum* biomass using response surface methodology.

**DOI:** 10.1016/j.dib.2020.105837

**Published:** 2020-06-08

**Authors:** Akeem Mohammed, Arianne Rivers, David.C. Stuckey, Keeran Ward

**Affiliations:** a Department of Chemical Engineering, University of the West Indies, St. Augustine, Trinidad and Tobago; b Department of Chemical Engineering, Imperial College London, London SW72AZ, UK

**Keywords:** Pelagic sargassum, Response surface methodology, Sodium alginate, Extraction, Optimization, Box-behnken design

## Abstract

This article presents data associated with the extraction of sodium alginate from waste *Sargassum* seaweed in the Caribbean utilizing an optimization approach using Response Surface Methodology [1]. A Box-Behnken (BBD) Response Surface Methodology using Design Expert 10.0.3 software on the alkaline extraction process was used. Data consists of the effects of 4 process variables (temperature, extraction time, alkali concentration and excess volume of alkali: dried seaweed) on the yield of sodium alginate. The model was validated, and extracts were characterization using High Performance Liquid Chromatography (HPLC), Gel Permeation Chromatography (GPC), Fourier Transform Infrared Spectroscopy (FTIR) and Nuclear Magnetic Resonance (NMR). The data illustrates the applicability of our model in potentially valorizing this waste product into a valuable resource. Furthermore, our methodology can be applied to other macroalgae for efficient extraction of sodium alginate of commercial quality.

Specifications Table

**Table:** 

**Subject area**	Chemical Engineering
**More specific subject area**	Extraction
**Type of data**	Tables and Figures
**How data was acquired?**	Specific ranges were considered among factors based on initial experiments. Design Expert 10.0.3 was used to formulate the experimental methodology, and to coordinate optimization through statistical verification. Sargassum biomass was harvested from the Trinidadian eastern coastal villages of Manzanilla and Mayaro, during May 2019.
**Data format**	Raw/Analyzed
**Parameters for Data Collection**	The main factors were temperature; 22–80 °C, alkali concentration; 1–10% (w/v) Na_2_CO_3_, alkali volume to dried seaweed ratio; 5–15 mL Na_2_CO_3_: 1 g dried seaweed and extraction time; 0.5–6 h.
**Description of Data collected**	Experimental yield and purity were determined using High Performance Liquid Chromatography (HPLC), over the experimental design. In addition, characterization of the raw *Sargassum* biomass (proximate analysis) and alginate polymers was carried out using Gel Permeation Chromatography (GPC), Fourier Transform Infrared Spectroscopy (FTIR), and H^1^ Nuclear Magnetic Resonance (NMR).
**Data source location**	Department of Chemical Engineering, University of the West Indies, St. Augustine, Trinidad, WI
**Data accessibility**	Repository Name: Mendeley Data; DOI: 10.17632/svn6c6zgx7.1 URL: https://data.mendeley.com/datasets/svn6c6zgx7/1
**Related research article**	Akeem Mohammed, Arianne Rivers, David. C. Stuckey and Keeran Ward, *Alginate Extraction from Sargassum Seaweed in the Caribbean Region: Optimization using Response Surface Methodology,* Carbohydrate Polymers, in press.

Value of the data

• This data can be used in combination with other datasets for developing future studies associated with alginate extraction from *Sargassum* biomass.
• This data can be extrapolated and adapted to solve optimization problems associated with inefficient extraction processes.
• The data can be used for comparison purposes with alginate extraction from other sources of macroalgae.
• The data serves as a basis for a waste-to-resource platform aimed at viable valorization of *Sargassum* within the Caribbean Region, currently experiencing the negative effects of this invasive species.

## 1 Data description

**Fig. 1:**
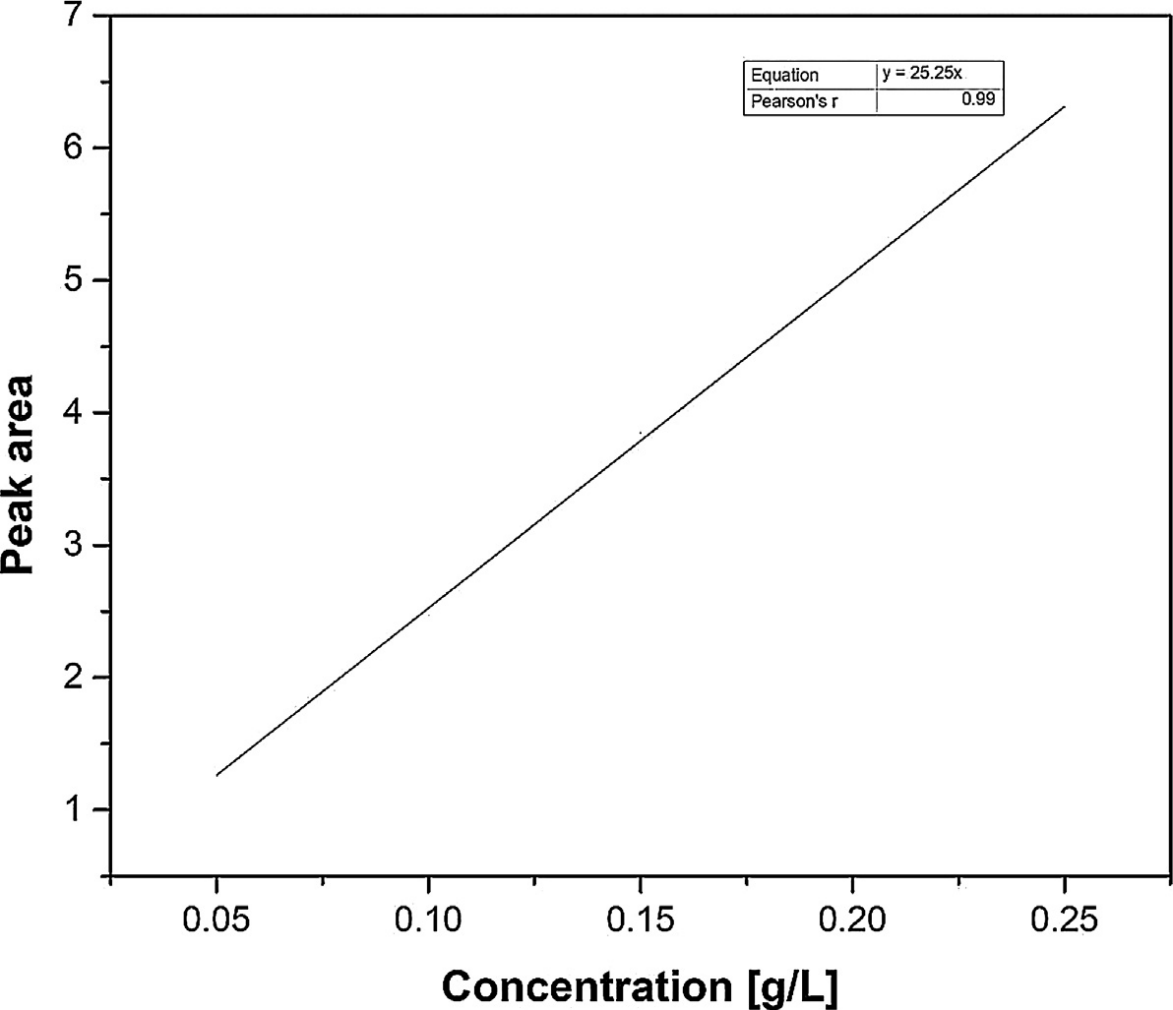
Calibration curve for HPLC analysis.

**Fig. 2:**
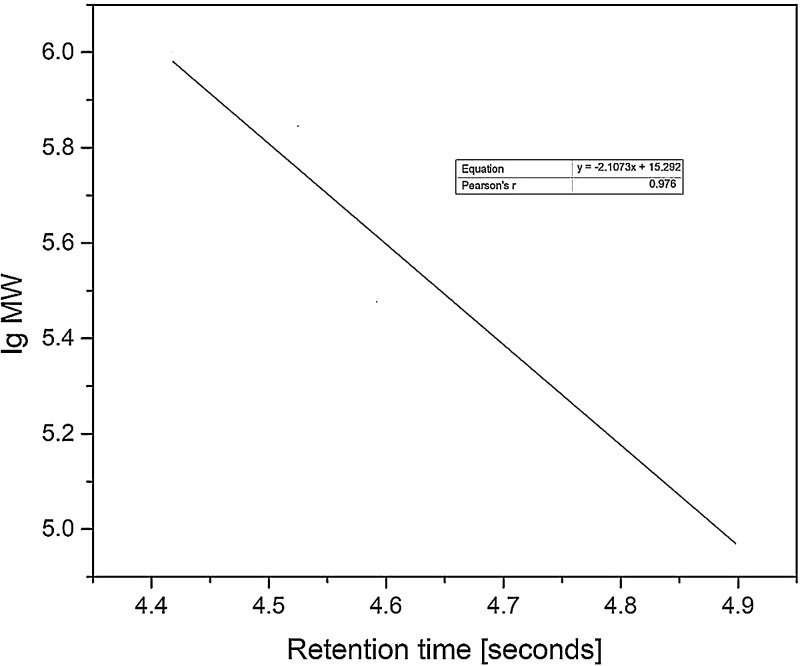
GPC calibration of Ln MW (molecular weight) against retention time.

The datasets illustrated here give characterized polymer concentrations from extracts determined over the design space from a total of 29 runs. Table 1 gives the crude polymer concentrations derived from the experimental methodology composed by Design of Experiments. Predicted concentrations generated based on experimental validation are shown in Table 2. Further isolation of the alginate polymer through purification using bleaching is given in Table 3. Product quality assurance measurements utilizing color measurements were also considered as shown in Table 4. Product characterization was as follows: polymer concentrations were determined using HPLC (Fig. 1) while GPC (Fig. 2) was used for molecular weight estimation. The signal intensities taken from NMR characterization obtained for different alginate samples, and calculated parameters for the alginate uronic acid sequences are given in Table 5. Supplementary data, available from DOI: 10.17632/svn6c6zgx7.1, gives the raw datasets compiled from extraction, optimization, characterization and quality assurance experimental methodologies comprising the multistage extraction process.

**Table 1:** HPLC data for 29 alginate extracts acquired over the Design Space. Specific Experimental Design is given in [1].

**Run #**	**Dilution factor**	**Area**	**Conc, mg/ml**
1	100	1.514	0.061
2	20	4.061	0.165
3	40	2.546	0.103
4	200	1.521	0.062
5	40	2.920	0.118
6	100	2.260	0.092
7	40	2.449	0.099
8	50	2.416	0.098
9	200	2.690	0.109
10	40	4.640	0.188
11	40	1.775	0.072
12	100	1.796	0.073
13	100	3.085	0.125
14	400	1.378	0.056
15	200	1.164	0.047
16	20	3.665	0.149
17	100	1.739	0.070
18	80	3.667	0.149
19	10	2.447	0.099
20	10	1.427	0.058
21	20	3.906	0.158
22	100	1.697	0.069
23	40	2.067	0.084
24	200	1.680	0.068
25	40	1.730	0.070
26	200	1.692	0.069
27	10	5.992	0.243
28	20	2.096	0.085
29	20	2.052	0.083

**Table 2:** HPLC data for the multistage (2 stages) extraction at determined optimum conditions.

**Run #**	**Dilution factor**	**Area**	**Conc, mg/ml**
**Stage 1**			
1	200	1.969	0.081
2	200	1.960	0.080
3	200	2.115	0.087
**Stage 2**			
1	40	3.552	0.145
2	40	3.439	0.141
3	40	3.764	0.154
4	40	3.722	0.152
5	40	3.610	0.148

**Table 3:** HPLC data for purity of bleached and unbleached alginate.

**Run #**	**Dilution factor**	**Area**	**Conc, mg/ml**
**Bleached**			
1	1	4.273	0.183
2	1	4.299	0.184
3	1	4.228	0.181
**Unbleached**			
1	1	4.279	0.183
2	1	4.350	0.186

**Table 4:** Data used for color analysis.

	**Alginate**		
**Parameter**	**Bleached**	**Unbleached**	**Food grade**
L	79.08	51.65	92.05
a	1.75	5.11	−0.06
b	12.55	8.95	7.91

**Table 5:** Signal intensities taken from the NMR spectra obtained for different alginate samples and calculated parameters for the uronic acid sequence.

**Signal**	**Food grade**	**Unbleached**	**Bleached**
A	0.0159	0.0119	0.0057
B1	0.0016	0.0007	0.0003
B2	0.0045	0.0008	0.0003
B3	0.0058	0.0016	0.0008
B4	0.0118	0.0044	0.0019
C	0.0079	0.0109	0.0052
G	0.0149	0.0122	0.0058
M	0.0178	0.0060	0.0026
GG	0.0089	0.0106	0.0051
MG	0.0060	0.0016	0.0007
MM	0.0118	0.0044	0.0019
GGM	0.0016	0.0007	0.0004
MGM	0.0044	0.0008	0.0004
GGG	0.0074	0.0099	0.0048

## 2 Experimental design, materials, and methods

### 2.1 Materials

Seaweed was collected from both Manzanilla and Mayaro bays on the Eastern Atlantic side of Trinidad in May 2019 and stored at −10 °C. The seaweed pre-treatment process was carried out using formaldehyde (BDH, 36.5 wt% in water). Acid treatment was conducted using sulphuric acid (J.T. Baker, 96.4%). For the alkaline extraction process, sodium carbonate (Scharlau, 99.9%) was used. Food grade sodium alginate (purity ≥ 96%) purchased from WillPowder (44145S, USA) was used as the analytical standard for High Performance Liquid Chromatography (HPLC). The buffer solution used for HPLC was made using phosphoric acid (J.T. Baker, purity > 86%) and sodium hydroxide (J.T Baker, >99%). Bleaching was done using sodium hypochlorite (Alfa Aesar, 11–15% available chlorine). For the purification process, 50% (w/v) alcohol (BDH, 94–96% ethanol and methanol) was used. H^1^ Nuclear Magnetic Resonance (NMR) analysis utilizing deuterium oxide (Sigma-Aldrich, purity >99.9%), triethylenetetraminehexaacetic acid (TTHA) (Sigma Aldrich, purity ≥ 98%) and sodium deuteroxide (Sigma-Aldrich, purity 99%) was used for characterization purposes.

### 2.2 Extraction of alginate and experimental design

Seaweed pre-treatment and acid treatment were done according to methods in our previous work [2]. Alkaline extraction was carried out over a temperature range 22–80 °C, a concentration range of 1–10% w/v Na_2_CO_3_, an excess volume range of 5–15 mL (Na_2_CO_3_: seaweed) and at reaction times ranging from 0.5–6 h. Box-Behnken experimental design (BBD) was chosen to investigate the effects of the aforementioned factors on extraction yield giving 29 experimental runs [1]. This crude yield was found using High Performance Liquid Chromatography (HPLC), with concentrations presented in Table 1.

### 2.3 Multistage extraction

Multistage extraction was carried out using methods derived in our previous work [2]. Optimum conditions were determined and validated in our study [1] using Derringer's desirability function found in Design Expert. Model validation was carried out at the optimum conditions and concentrations are presented in Table 2.

### 2.4 Bleaching and precipitation of alginate

The purity of bleached and unbleached alginate samples was found using HPLC by comparing the alginate extracted to that of a commercial standard sample of concentration 0.2 g/ml (Table 3).

### 2.5 Color analysis

Color measurements were carried out on the purified alginate powder, and the Whiteness Index (WI) determined using the Hunter (L, a, b) color measurement system [3]. The equation used is available [1]. The dataset for the color analysis is presented in Table 4.

### 2.6 Characterization

#### 2.6.1 HPLC

The HPLC methodology was adapted from Awad and Aboul-Enein [4]. Alginate standards were made utilizing a 1 g/L analytical sodium alginate solution, within the calibration range of 0.05–0.25 g/L. The calibration curve and equation is presented in Fig. 1.

#### 2.6.2 GPC

Polyethylene oxide (PEO) standards (100 −1000 kDa, Sigma Aldrich, Switzerland) was used as adapted from Kapishon, Whitney [5]. A calibration curve (Fig. 2) of Ln MW (molecular weight) against retention time was obtained.

#### 2.6.3 NMR

NMR analysis was carried out according to ASTM F2259-10 [6]. The chemical shifts of the anomeric proton signals were A (guluronic acid anomeric proton) at around 5.08 ppm; B1 (H-5 proton of the central guluronic acid residue in a GGM triad) at 4.76 ppm; B2 (H-5 proton of the central guluronic acid residue in a MGM triad) at 4.73 ppm; B3 (anomeric proton of the mannuronic acid residue neighboring a mannuronic acid) at 4.70 ppm; B4 (anomeric proton of the mannuronic acid residue neighboring a guluronic acid) at 4.68 ppm and C (guluronic acid proton 5) at 4.48 ppm. The signals from the NMR spectra are given in Table 5. The following equations were used to determine the uronic acid sequence [6]:

(1) G=0.5(A+C+0.5(B1+B2+B3))

(2) M=B4+0.5(B1+B2+B3)

(3) GG=0.5(A+C−0.5(B1+B2+B3))

(4) MG=GM=0.5(B1+B2+B3)

(5) MM=B4

(6) $$GGM=MGG=\frac{\left(B1\right)\left(0.5\right)\left(B1+B2+B3\right)\;\;\;\;}{\left(B1+B2\right)}$$

(7) $$MGM=\frac{\left(B2\right)\left(0.5\right)\left(B1+B2+B3\right)\;\;\;\;}{\left(B1+B2\right)}$$

(8) GGG=GG−GGM

## Declaration of Competing Interest

The authors declare that they have no known competing financial interests or personal relationships that could have appeared to influence the work reported in this paper.
